# Overexpression of *SbMYB38* from *Sorghum bicolor* Enhances Saline–Alkali Stress Tolerance in *Arabidopsis thaliana*

**DOI:** 10.3390/plants15213283

**Published:** 2026-10-24

**Authors:** Xiaoxue Li, Kaifeng Yang, Lan Wei, Jiaxin Yang, Mi Zhang, Hongcheng Wang

**Affiliations:** School of Life Sciences, Guizhou Normal University, Guiyang 550025, China; 242100100432@gznu.edu.cn (X.L.); 252200101667@gznu.edu.cn (K.Y.); 242100100431@gznu.edu.cn (L.W.); 252200101658@gznu.edu.cn (J.Y.); 2025100100425@gznu.edu.cn (M.Z.)

**Keywords:** saline–alkali stress, sorghum, *SbMYB38*, transcriptome, flavonoids

## Abstract

Saline–alkali stress is a major environmental factor limiting plant growth and development, primarily because it causes ion toxicity, oxidative damage, and osmotic stress. Studies have shown that some members of the MYB gene family in *Sorghum bicolor* can enhance plant tolerance to saline–alkali stress. To clarify the specific function of the MYB family member *SbMYB38* under saline–alkali stress, we cloned the coding sequence of *SbMYB38* and successfully transformed it into *Arabidopsis thaliana*. Subcellular localization results showed that *SbMYB38* was located in the nucleus. Overexpression of *SbMYB38* in transgenic *Arabidopsis thaliana* (OE) increased plant tolerance to saline–alkali stress. After treatment with 150 mM NaHCO_3_, the activities of the antioxidant enzymes superoxide dismutase (SOD), peroxidase (POD), and catalase (CAT) were significantly higher in the OE lines than in the wild type (WT). Under 150 mM NaHCO_3_ stress, the expression levels of genes related to the absorption and transport of protons, sodium ions, and potassium ions, such as *AtAHA2*, *AtSOS1* and *AtNHX1*, showed different changes. Physiological and biochemical analyses indicated that following NaHCO_3_ treatment, the transgenic lines had significantly increased proline and chlorophyll contents and decreased malondialdehyde (MDA) content. In addition, transcriptomic data showed that the key genes involved in flavonoid biosynthesis, including *FLS*, *4CL3*, and *CYP75B1*, were differentially upregulated, whereas key genes involved in lignin biosynthesis, such as *HCT*, *CCoAOMT*, *CAD*, and *CCR*, were downregulated. This suggests that *SbMYB38* may enhance plant tolerance to saline–alkali stress by participating in flavonoid biosynthesis. However, this hypothesis requires further verification. Collectively, these findings provide a basis for understanding the molecular mechanisms of plant saline–alkali tolerance and offer valuable resources for breeding tolerant crops and for the phytoremediation of saline–alkali land.

## 1. Introduction

Saline–alkali stress is one of the major abiotic stress factors affecting normal growth of crops [1]. Data show that more than 800 million hectares of global farmland are affected by salinization. In China, saline–alkali land accounts for 25% of the total arable land area, which has seriously affected crop and agricultural production [2]. The main harmful effects of saline–alkali stress on plants include ion toxicity, oxidative damage, osmotic stress, and high-pH damage [3]. It damages plant cell membranes, reduces the activities of related enzymes, causes plant metabolic disorders and poor nutrient absorption, and leads to poor plant development, slow growth, and plant damage [4]. Therefore, elucidating the tolerance mechanisms of plants under saline–alkali stress and developing strategies to mitigate soil salinization and its damage to plants have become urgent problems that need to be solved.

When plants are under saline–alkali stress, they carry out a series of complex physiological and molecular mechanisms to resist the stress [5]. Saline–alkali stress causes Na^+^ to enter plant cells, resulting in ion toxicity; simultaneously, it creates an osmotic potential that causes water inside the cells to flow out [6]. Plants expel Na^+^, and they also use ion antiporters such as *SOS1* in the cell membrane and *NHX1* in the vacuolar membrane to isolate Na^+^; they reduce the toxic effects in this way [7]. The H^+^-ATPase is located on the cell and vacuolar membranes. It generates a proton driving force, which drives the transport of Na^+^ in plants [8]. Saline–alkali stress increases the activity of H^+^-ATPase, causing more H^+^ ions to be pumped into the vacuole and increasing the transmembrane electrochemical gradient. Then Na^+^ efflux from the cell to the outside is enhanced, and osmotic balance is better maintained [9]. K^+^ concentration is closely related to osmotic regulation, membrane potential, and enzyme activity in plants [10,11]. Under saline–alkali stress, the entry of large amounts of Na^+^ disrupts the K^+^/Na^+^ balance. This disruption interferes with the normal absorption and transport of water in plants, affects the opening and closing of ion channels on cell membranes, and impairs energy metabolism and the plant’s ability to defend against oxidative stress [12]. However, the transcriptional regulatory networks governing these ion transport-related genes under saline–alkali stress remain to be fully elucidated.

Osmotic and ionic stresses also lead to the production of reactive oxygen species (ROS). Excessive ROS have strong oxidizing ability. They attack the unsaturated fatty acids on the plant cell membrane, triggering membrane lipid peroxidation. This damages the selective permeability and integrity of the membrane [13]. Meanwhile, they also cause protein carbonylation and DNA strand breakage, ultimately leading to programmed cell death in plants [14]. Furthermore, ROS affect the functional components of plant organelles and cause structural damage, subsequently leading to the occurrence of oxidative stress in plants [15]. To prevent the excessive production of reactive oxygen species, plants employ several enzymes as scavengers under saline–alkali stress, including superoxide dismutase (SOD), catalase (CAT), and peroxidase (POD). They work together to remove ROS [16]. In addition, secondary metabolites in plants, such as amino acids, sugars, pigments, flavonoids, and terpenoids, also possess antioxidant effects [17]. Among them, flavonoid compounds, as an important and large class of secondary metabolites, play a crucial role in this process [18].

MYB transcription factors constitute a common family of transcription factors in eukaryotes. Their core part is a highly conserved DNA-binding domain at the N-terminus of the protein. This domain contains 1 to 4 characteristic repeats. Based on sequence similarity, the MYB domain repeats can be divided into R1, R2, or R3 types [19]. Each repeat (R) consists of 51 to 52 conserved amino acids and spacer sequences. There is a tryptophan residue every 18 amino acids, which is crucial for maintaining the hydrophobic nature of the domain [20]. MYB transcription factors can be divided into four main categories, namely 1R-MYB, R1R2-MYB, 3R-MYB, and 4R-MYB [21]. In existing studies, most MYB transcription factors play either an activating or inhibiting role, and they can participate in regulating plant life activities and various metabolic processes [22]. Under abiotic stress, MYB transcription factors are involved in functions such as regulating flavonoid and cuticle synthesis, responding to drought stress, and mediating plant responses to biotic and abiotic stresses [23]. For example, *MYB12* upregulates the expression of the early flavonol biosynthesis genes chalcone synthase (CHS) and flavonol synthase (FLS). In transgenic plants, overexpression of *MYB12* together with *MYB75* significantly increases the accumulation of flavonoids with strong antioxidant activity and enhances plant tolerance to abiotic stresses such as drought stress and oxidative stress [24]. In sorghum, the expression of *SbMYBAS1* is downregulated under saline–alkali stress, and its overexpression reduces the salt tolerance of *Arabidopsis thaliana* [23]. The *TaMYBsdu1* gene in wheat plays an important role in responding to saline–alkali and drought stresses [25]. *OsMYB2* in rice responds to salt and drought stresses [26]. Overexpression of *TaMYB344* in transgenic tobacco improved its tolerance to drought, high temperature, and high saline–alkali stress [27]. Currently, there are many reviews on *MYB* genes, but there is a lack of functional validation for *MYB38*.

In our previous study, we sequenced the transcriptome of *Sorghum bicolor* under saline–alkali stress and found that the *MYB38* gene was significantly upregulated [28]. However, the role of this gene in the physiological response and molecular mechanism under sodium bicarbonate saline–alkali stress is still unclear. For this purpose, the *SbMYB38* gene was successfully overexpressed in *Arabidopsis thaliana* to verify the molecular mechanism under saline–alkali stress. Overexpression of *SbMYB38* in transgenic *Arabidopsis thaliana* (OE) can effectively alleviate the toxicity caused by saline–alkali stress. According to the qRT-PCR results, the expression of genes related to proton and Na^+^ transport was upregulated in the OE lines, while genes related to K^+^ export were downregulated. In addition, the RNA-seq data suggest that overexpression of *SbMYB38* may enhance the tolerance of *Arabidopsis thaliana* to saline–alkali stress by regulating flavonoid synthesis.

## 2. Results

### 2.1. Phylogenetic Analysis, Multiple Sequence Alignment, and Conserved-Domain Characterization of SbMYB38 and Its Homologous Proteins

**Figure 1:**
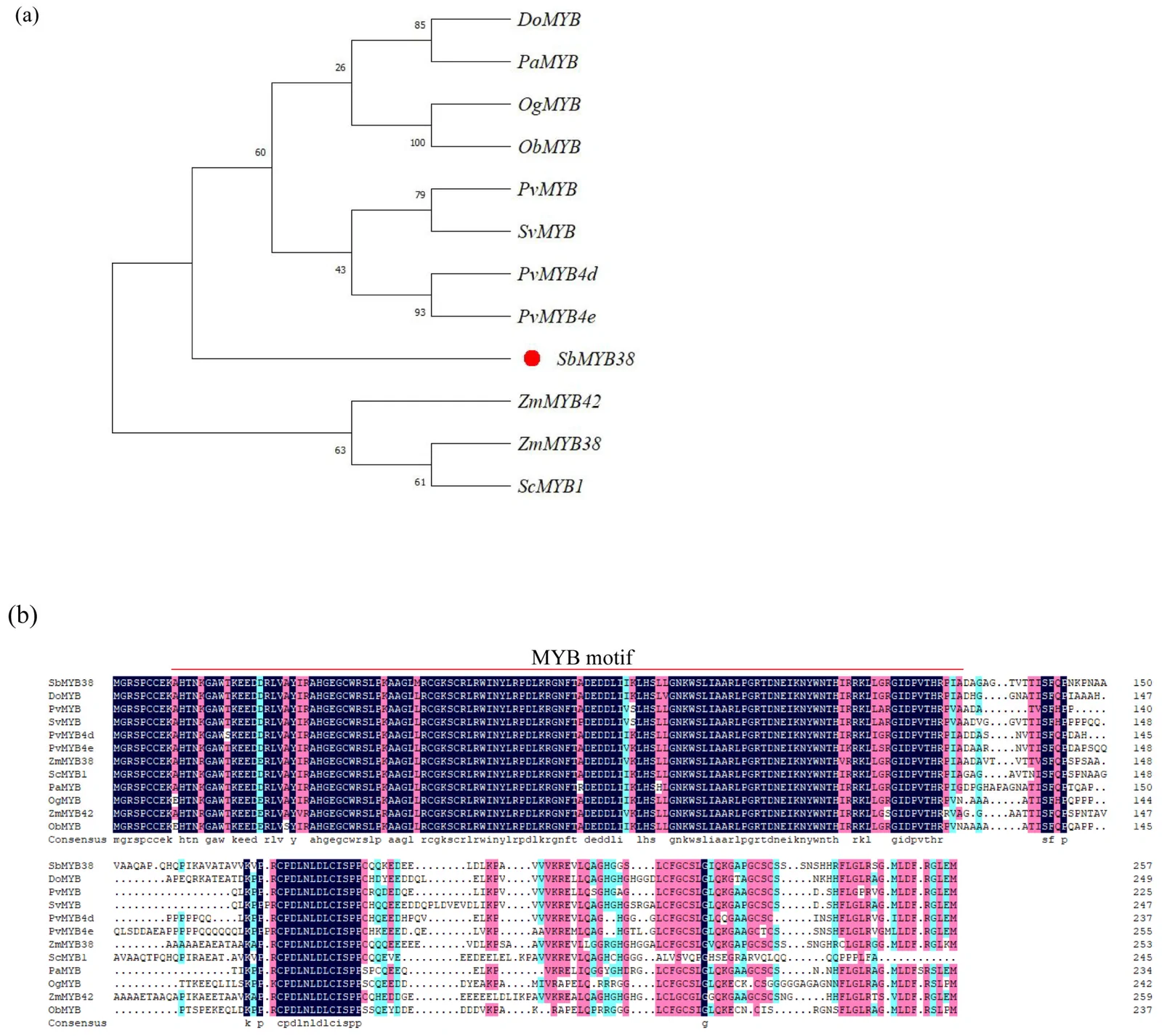
Phylogenetic relationships of SbMYB38 with other plant homologous proteins (**a**) and multiple sequence alignment (**b**). The red lines indicate the MYB domains.

The SbMYB38 protein is conserved among nine plant species. Sequence alignment showed that the domain of SbMYB38 is identical to that in other plants (Figure 1b). To further understand the phylogenetic position of these nine proteins, phylogenetic analysis of the protein sequences was performed using MEGA7.0 (Molecular Evolutionary Genetics Analysis, Sudhir Kumar, Tempe, Arizona, USA). SbMYB38 is evolutionarily close to the protein from *Zea mays* (Figure 1a). Multiple sequence alignment of the conserved domains showed that the MYB domains of SbMYB38 are similar to those of MYB proteins from other plant species.

### 2.2. SbMYB38 Is Localized in the Nucleus

**Figure 2:**
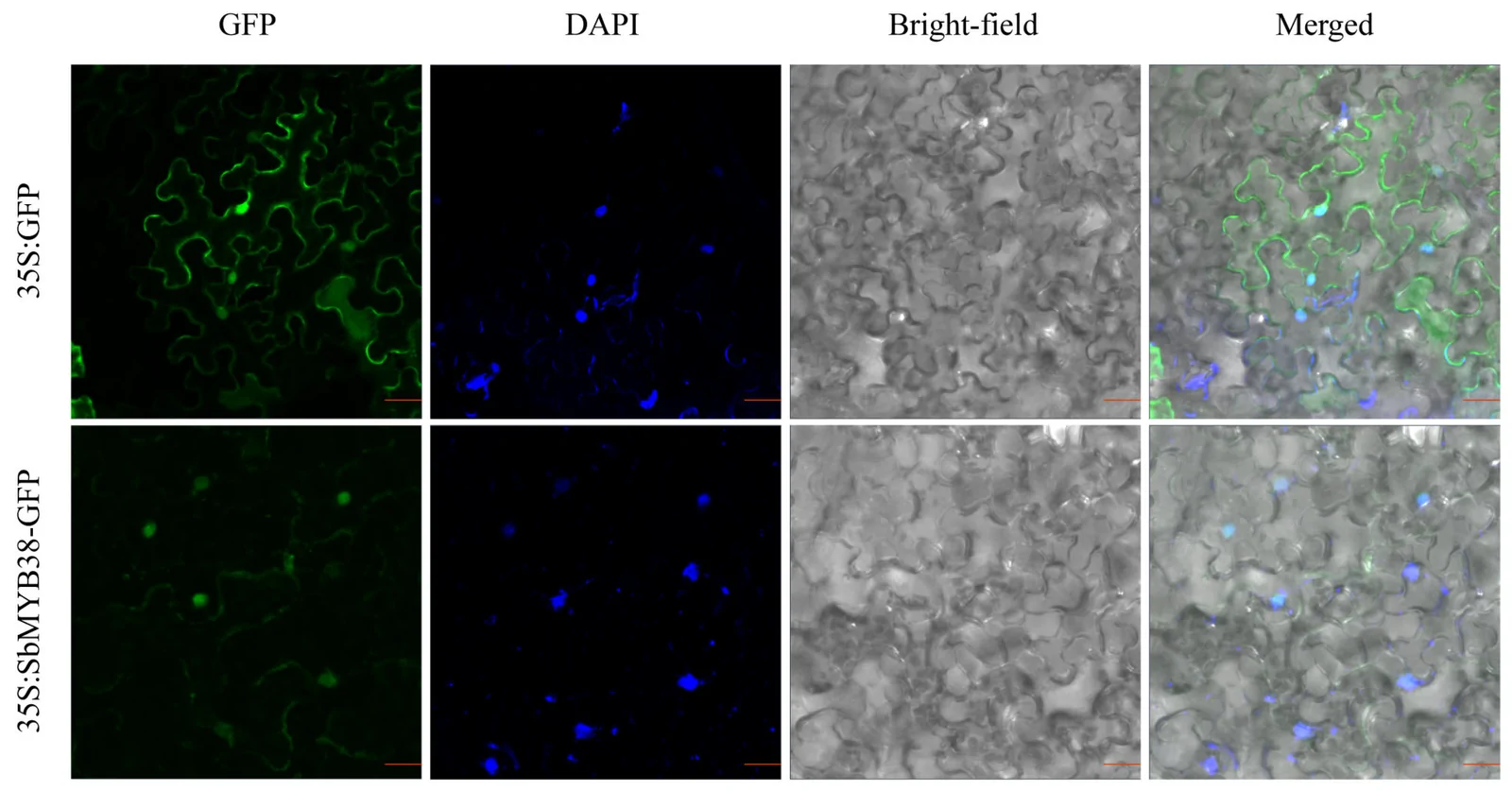
Subcellular localization of SbMYB38 in the nucleus. Note: GFP: green fluorescence; Bright-field: bright field; Merged: merged field. Scale bar = 50 μm.

To determine where the SbMYB38 protein is located inside plant cells, the SbMYB38-GFP and GFP expression vectors were injected into tobacco epidermal cells for transient expression. The green fluorescent signal of the SbMYB38-GFP fusion protein appeared as dots, and this signal overlapped with the nuclear region stained by DAPI, indicating that the SbMYB38 protein is mainly distributed in the nucleus of plant cells (Figure 2).

### 2.3. Saline–Alkali Stress Induces SbMYB38 Expression

**Figure 3:**
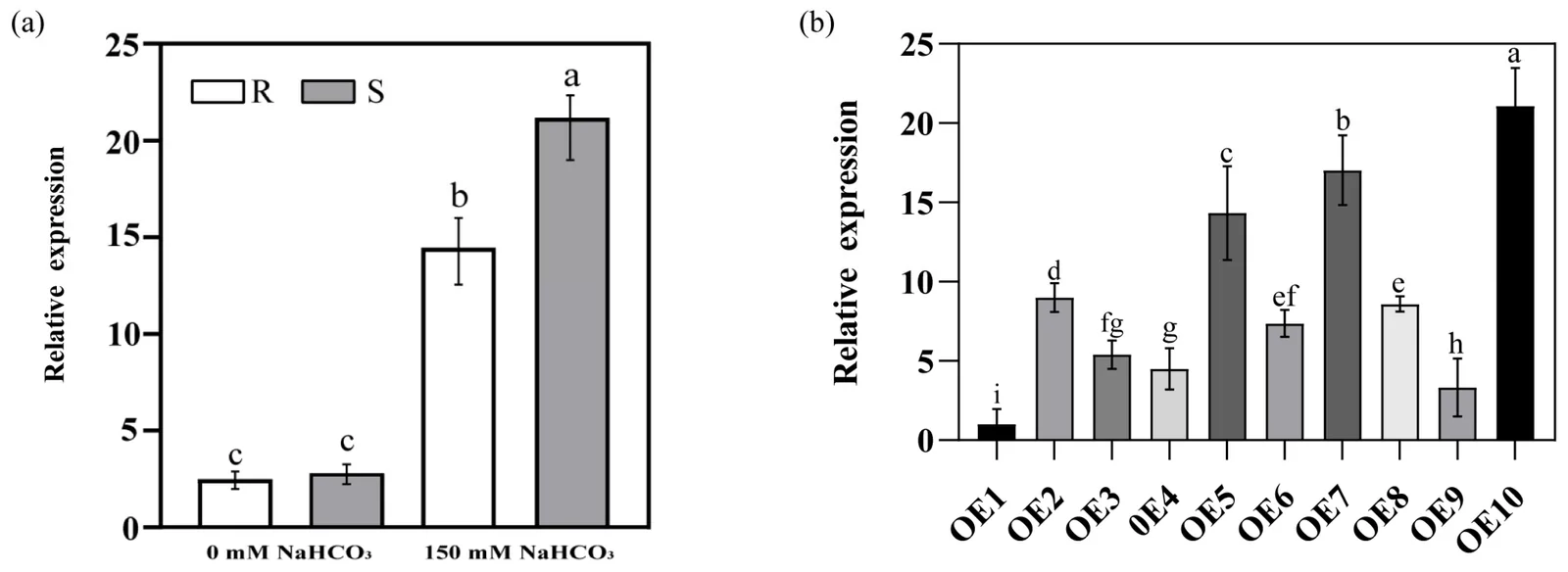
Expression patterns of the *SbMYB38* gene. (**a**) Relative expression of *SbMYB38* in *Sorghum bicolor* under 150 mM NaHCO_3_ stress. S, shoot; R, root. (**b**) Expression of *SbMYB38* in transgenic *Arabidopsis thaliana*. Data are presented as the mean ± standard deviation (SD) of three biological replicates. Different lowercase letters above the bars indicate significant differences among the groups (*p* < 0.05). This figure was plotted using GraphPad Prism 10; the significance analysis for letter labeling was performed using one-way analysis of variance followed by Duncan’s multiple range test, completed with IBM SPSS Statistics 20.

To investigate the response characteristics of the *SbMYB38* gene to saline–alkali stress, real-time quantitative PCR (qRT-PCR) was performed. The results showed that under normal conditions, SbMYB38 expression did not differ significantly between roots and shoots. However, after treatment with 150 mM NaHCO_3_, its expression was significantly upregulated (Figure 3a), indicating that this gene is induced by saline–alkali stress. Meanwhile, we constructed an *SbMYB38* overexpression vector and transformed it into *Arabidopsis thaliana* to obtain stable transgenic lines. qRT-PCR analysis revealed that the expression level of *SbMYB38* in transgenic *Arabidopsis* was significantly higher than that in the wild type (Figure 3b). We selected three transgenic lines, OE5, OE7, and OE10, which exhibited relatively higher expression levels, as experimental materials for subsequent experiments.

### 2.4. SbMYB38 Overexpression Improves NaHCO_3_ Tolerance in Transgenic Arabidopsis thaliana

**Figure 4:**
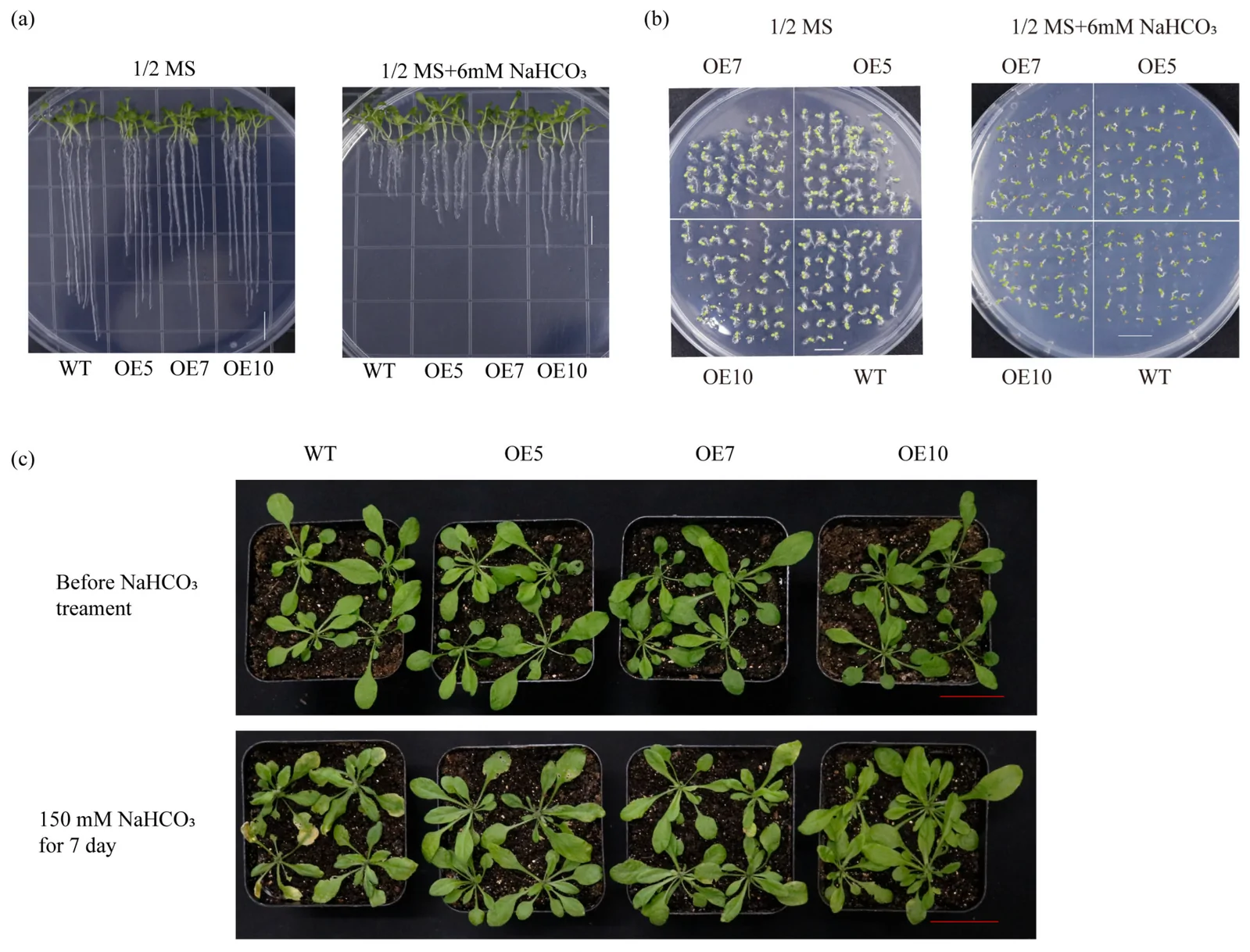
Growth status of *Arabidopsis* overexpressing the *SbMYB38* gene under saline–alkali stress. (**a**,**b**) Root length and germination rate of transgenic *Arabidopsis* grown on 1/2 MS medium without NaHCO_3_ and on 1/2 MS medium containing 6 mM NaHCO_3_ for 7 days. Scale bar: 1 cm. (**c**) Shoot morphology of wild-type (WT) and transgenic *Arabidopsis* treated with 150 mM NaHCO_3_ for 7 days. Note: Scale bar = 4 cm.

**Figure 5:**
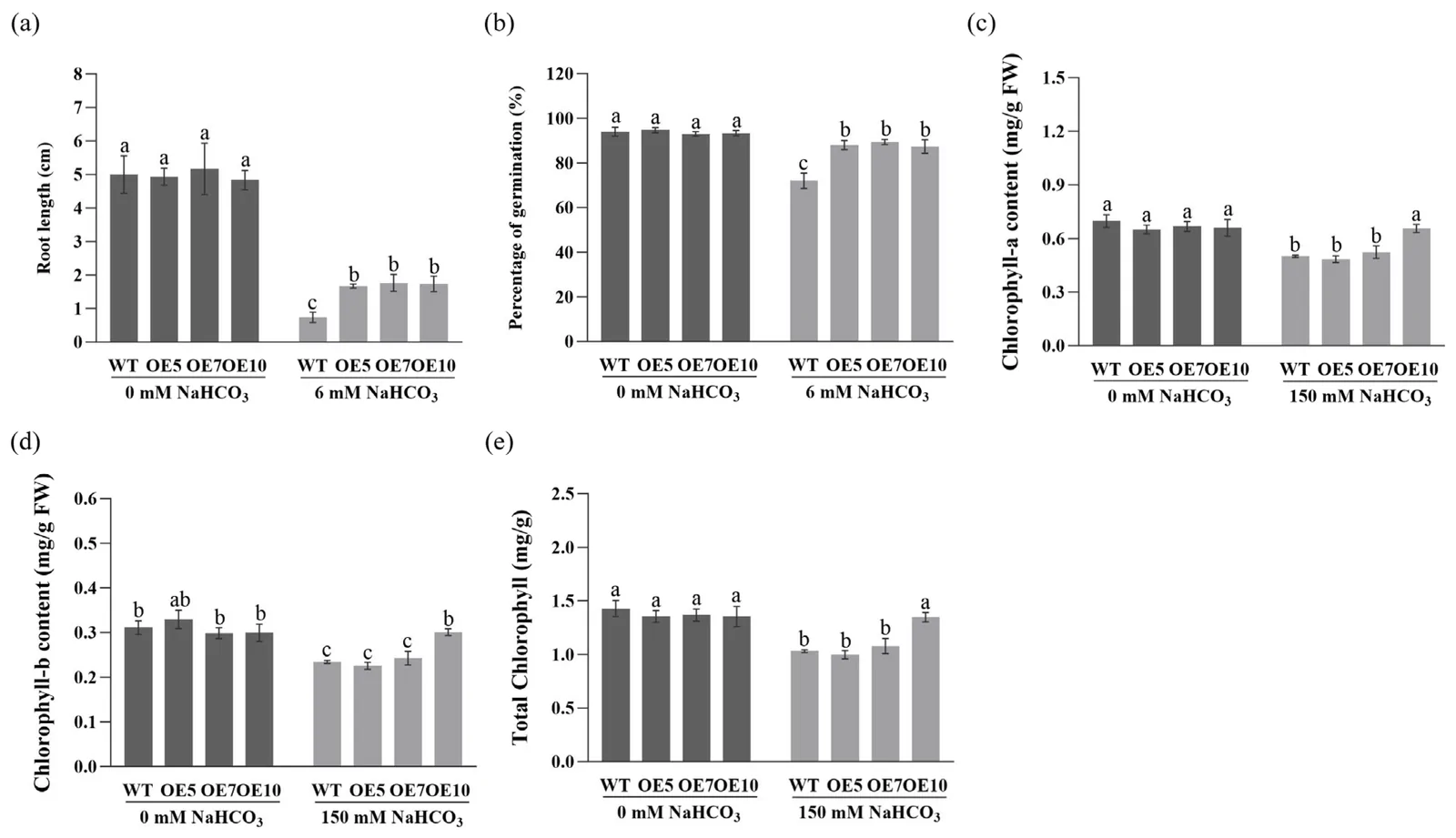
Determination of root length, germination rate, and chlorophyll content in wild-type and transgenic *Arabidopsis* overexpressing the *SbMYB38* gene under saline–alkali stress. (**a**) Root length. (**b**) Germination percentage. (**c**) Chlorophyll a. (**d**) Chlorophyll b. (**e**) Total chlorophyll. Data are presented as the mean ± standard deviation (SD) of three biological replicates. Different lowercase letters above the bars indicate significant differences among the groups (*p* < 0.05). This figure was plotted using GraphPad Prism 10; the significance analysis for letter labeling was performed using one-way analysis of variance followed by Duncan’s multiple range test, completed with IBM SPSS Statistics 20.

To further evaluate the functional role of *SbMYB38* in saline–alkali stress tolerance, we compared the phenotypic responses of transgenic lines and wild-type (WT) plants under stress conditions. Under normal growth medium, there was no significant difference in root length and germination rate between the wild type and the three transgenic lines. However, after treatment with 6 mM NaHCO_3_ for 7 days, the root length of the wild type was significantly shorter than that of the three transgenic lines (Figure 4a and Figure 5a). The germination rate decreased in both the wild type and the transgenic lines, but the decrease was more significant in the wild type (Figure 4b and Figure 5b). After 14 days of soil culture, under normal conditions, there was no difference in phenotype between the wild type and the transgenic lines. After treatment with 150 mM NaHCO_3_ for 7 days, the wild type showed severe leaf yellowing and wilting, and some even died. In contrast, the transgenic lines maintained better growth under stress and did not show obvious growth inhibition (Figure 4c). These results indicate that *SbMYB38* can improve the tolerance of *Arabidopsis* to saline–alkali stress. Under saline–alkali stress, the chlorophyll content in the three transgenic lines was less damaged and was significantly higher than that in the wild-type plants (Figure 5c–e). These results indicate that overexpression of *SbMYB38* in *Arabidopsis* enhances tolerance to saline–alkali stress by increasing antioxidant capacity and rescuing photosynthesis.

### 2.5. SbMYB38 Enhances Saline–Alkali Tolerance in Transgenic Arabidopsis Thaliana by Increasing Antioxidant Enzyme Activities and Proline Content

**Figure 6:**
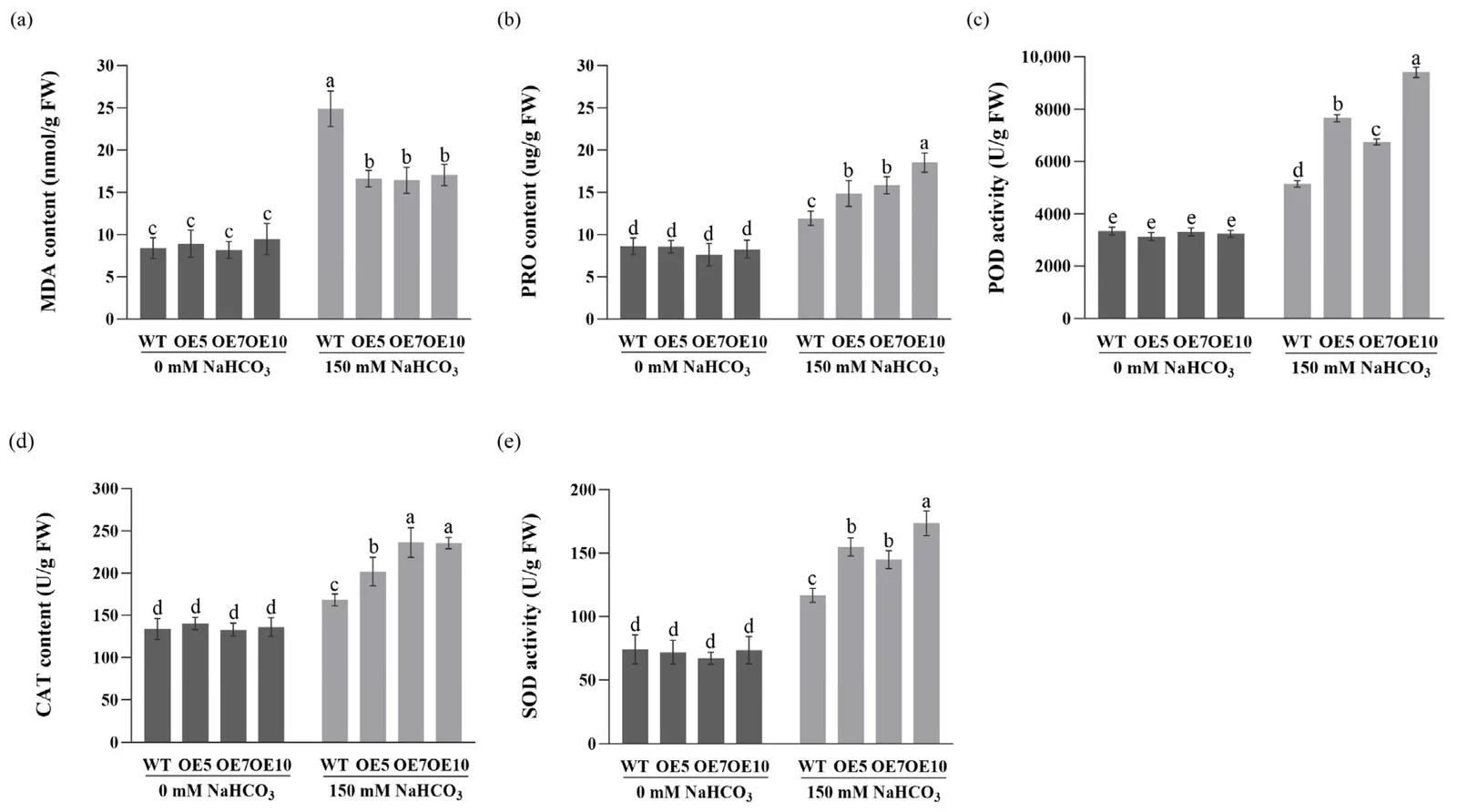
Physiological indices of plants expressing *SbMYB38* under control and 150 mM NaHCO_3_ stress conditions. (**a**) Malondialdehyde (MDA) content. (**b**) Proline content. (**c**) Peroxidase (POD) activity. (**d**) Catalase (CAT) activity. (**e**) Superoxide dismutase (SOD) activity. Data are presented as the mean ± standard deviation (SD) of three biological replicates. Different lowercase letters above the bars indicate significant differences among the groups (*p* < 0.05). This figure was plotted using GraphPad Prism 10; the significance analysis for letter labeling was performed using one-way analysis of variance followed by Duncan’s multiple range test, completed with IBM SPSS Statistics 20.

**Figure 7:**
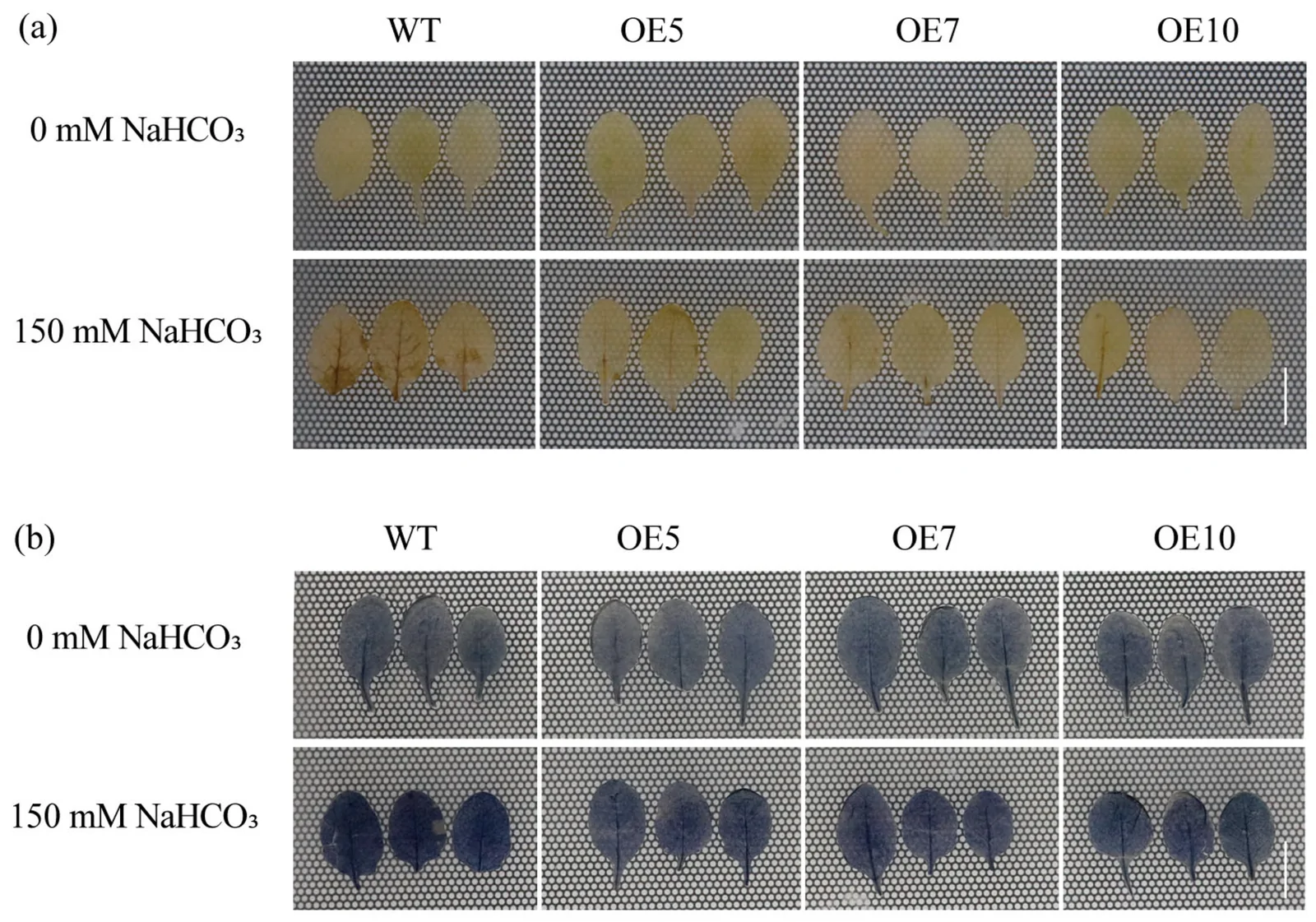
Histochemical staining of H_2_O_2_ and O_2_^−^ accumulations in WT and *SbMYB38*-transgenic *Arabidopsis* under saline–alkali stress. (**a**) DAB staining. (**b**) NBT staining. Plants of each line were grown in soil for 14 days, with or without 150 mM NaHCO_3_ supplementation. Scale bar = 1 cm.

We measured the content of malondialdehyde (MDA) and proline (PRO), which can reflect the degree of damage to plants under stress (Figure 6a,b). The results showed that under normal growth conditions, the MDA and Pro content in all lines remained at a certain level, and there was no significant difference among the lines. However, after saline–alkali treatment, the MDA content increased in all lines to different extents. The accumulation of MDA in the three overexpression lines was significantly lower than that in the wild type. Compared with the transgenic plants, the proline content in the wild type showed a decreasing trend. These two indicators suggest that the cell membranes of wild-type plants were more severely damaged. At the same time, the antioxidant system in the plants began to function, reducing the pressure of saline–alkali stress by removing some reactive oxygen species. The activities of peroxidase (POD), superoxide dismutase (SOD), and catalase (CAT) in the three transgenic lines were significantly higher than those in the wild-type plants (Figure 6c–e). DAB and NBT staining showed that under saline–alkali stress, the levels of H_2_O_2_ and O_2_^−^ in the wild-type lines were higher than those in the overexpression lines (Figure 7a,b). These results indicate that overexpression of *SbMYB38* in *Arabidopsis* enhances tolerance to saline–alkali stress by increasing antioxidant capacity and rescuing photosynthesis.

### 2.6. SbMYB38 Overexpression Affects the Expression Levels of Saline–Alkali Stress-Related Genes

**Figure 8:**
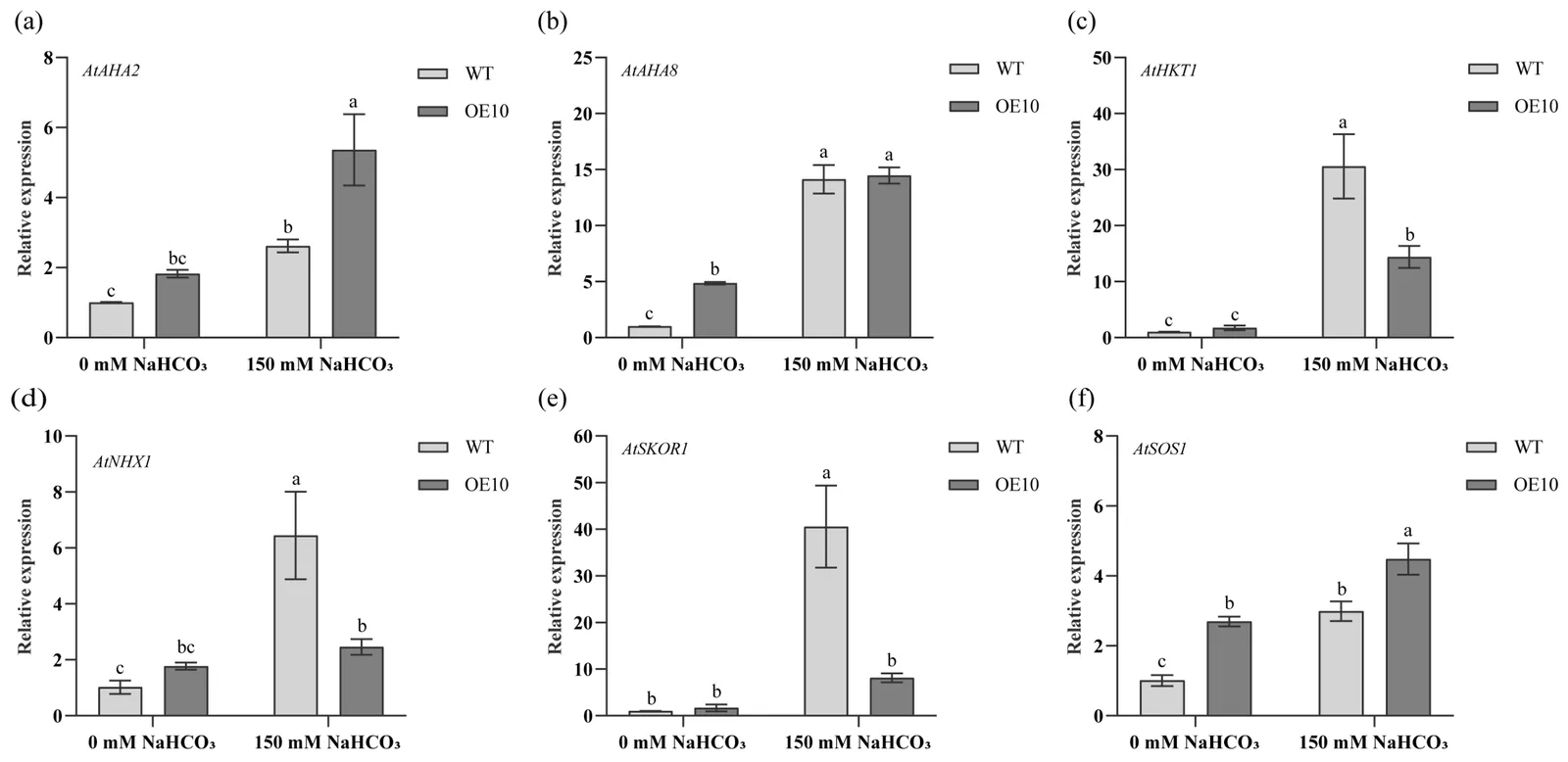
Relative expression levels of proton, sodium ion and potassium ion transporters (**a**–**f**) in wild-type and transgenic lines under 0 mM NaHCO_3_ and 150 mM NaHCO_3_ stress. (**a**–**f**) Relative expression levels of *AtAHA2*, *AtAHA8*, *AtAHKT1*, *AtNHX1*, *AtSKOR1* and *AtSOS1*. Data are presented as the mean ± standard deviation (SD) of three biological replicates. Different lowercase letters above the bars indicate significant differences among the groups (*p* < 0.05). This figure was plotted using GraphPad Prism 10; the significance analysis for letter labeling was performed using one-way analysis of variance followed by Duncan’s multiple range test, completed with IBM SPSS Statistics 20.

Using qRT-PCR, the expression of genes related to saline–alkali stress was examined in the *SbMYB38* overexpression lines. The results showed that under saline–alkali stress, the expression of H^+^-ATPase genes *AtAHA2* and *AtAHA8* was upregulated (Figure 8a,b). The expression of K^+^ transporter genes *AtHKT1*, *AtNHX1* and *AtSKOR1* was downregulated (Figure 8c–e). The expression level of the Na^+^ transporter gene *AtSOS1* was upregulated, and its expression in the overexpression lines was significantly higher than that in the wild-type lines (Figure 8f).

### 2.7. SbMYB38 Promotes the Expression of Flavonoid Pathway Genes in the Aerial Parts of Transgenic Plants

**Figure 9:**
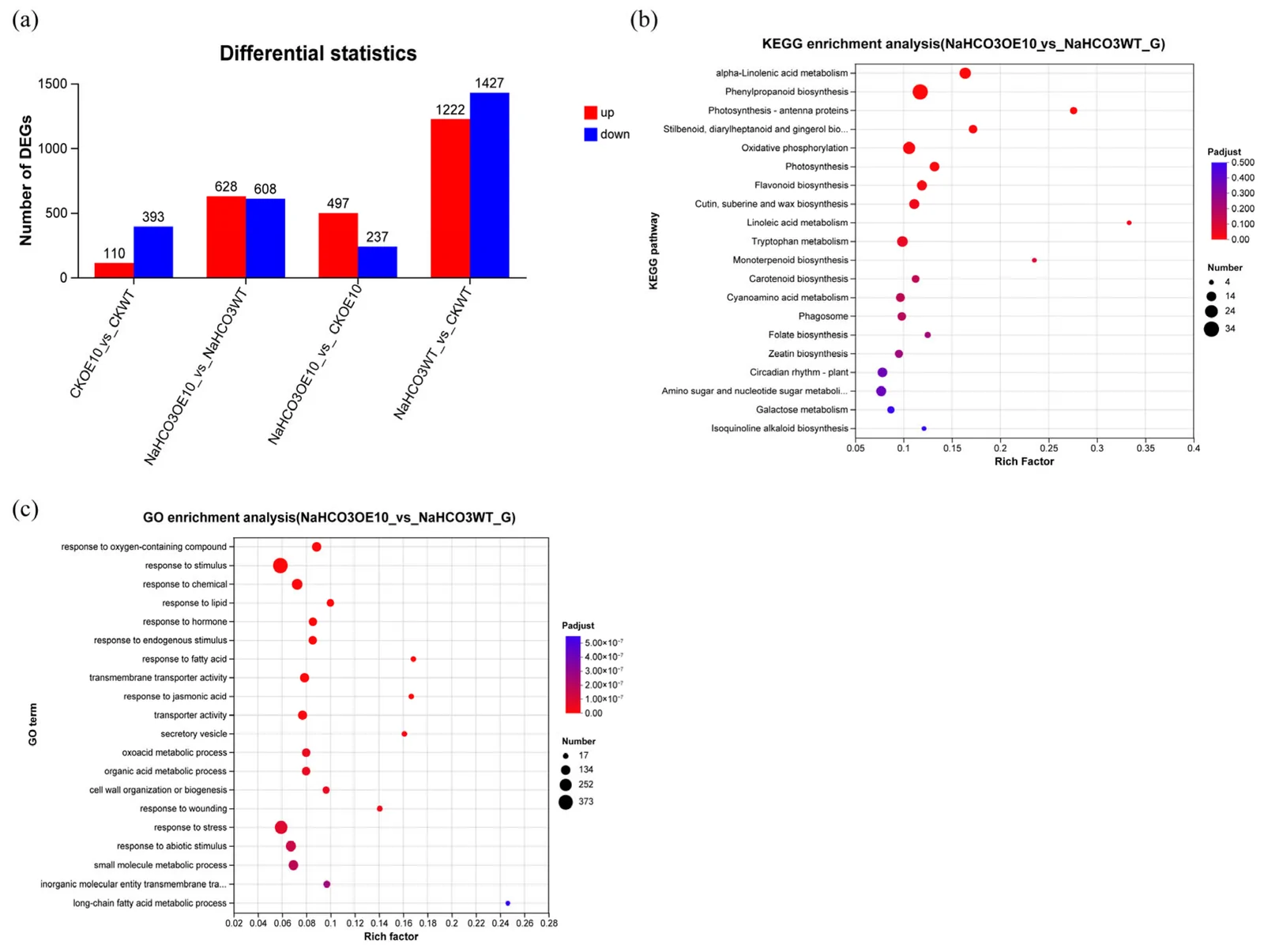
(**a**) Number of differentially expressed genes (DEGs) identified in the shoots of wild-type (WT) and *SbMYB38*-overexpressing transgenic line OE10 under saline–alkali stress through transcriptome analysis. (**b**) KEGG pathway enrichment of DEGs in the shoots of WT and OE10 plants affected by 150 mM NaHCO_3_ stress. (**c**) Gene Ontology (GO) assignment of DEGs in the shoots of WT and OE10 plants affected by 150 mM NaHCO_3_ stress.

To understand the regulatory network after introducing the *SbMYB38* gene into *Arabidopsis thaliana*, we treated the wild type (WT) and the OE10 line with 0 mM and 150 mM NaHCO_3_ and identified differentially expressed genes (DEGs) in their above-ground parts. There were 734 DEGs between the NaHCO_3_ -treated and untreated transgenic lines, and 2649 DEGs between the NaHCO_3_-treated and untreated WT lines. In addition, a total of 1236 DEGs were identified between the NaHCO_3_-treated WT and transgenic lines, while 503 DEGs were identified between the untreated WT and transgenic lines. These results indicate that the introduction of the *SbMYB38* gene into *Arabidopsis* changed the transcriptional profile in response to saline–alkali stress (Figure 9a).

**Figure 10:**
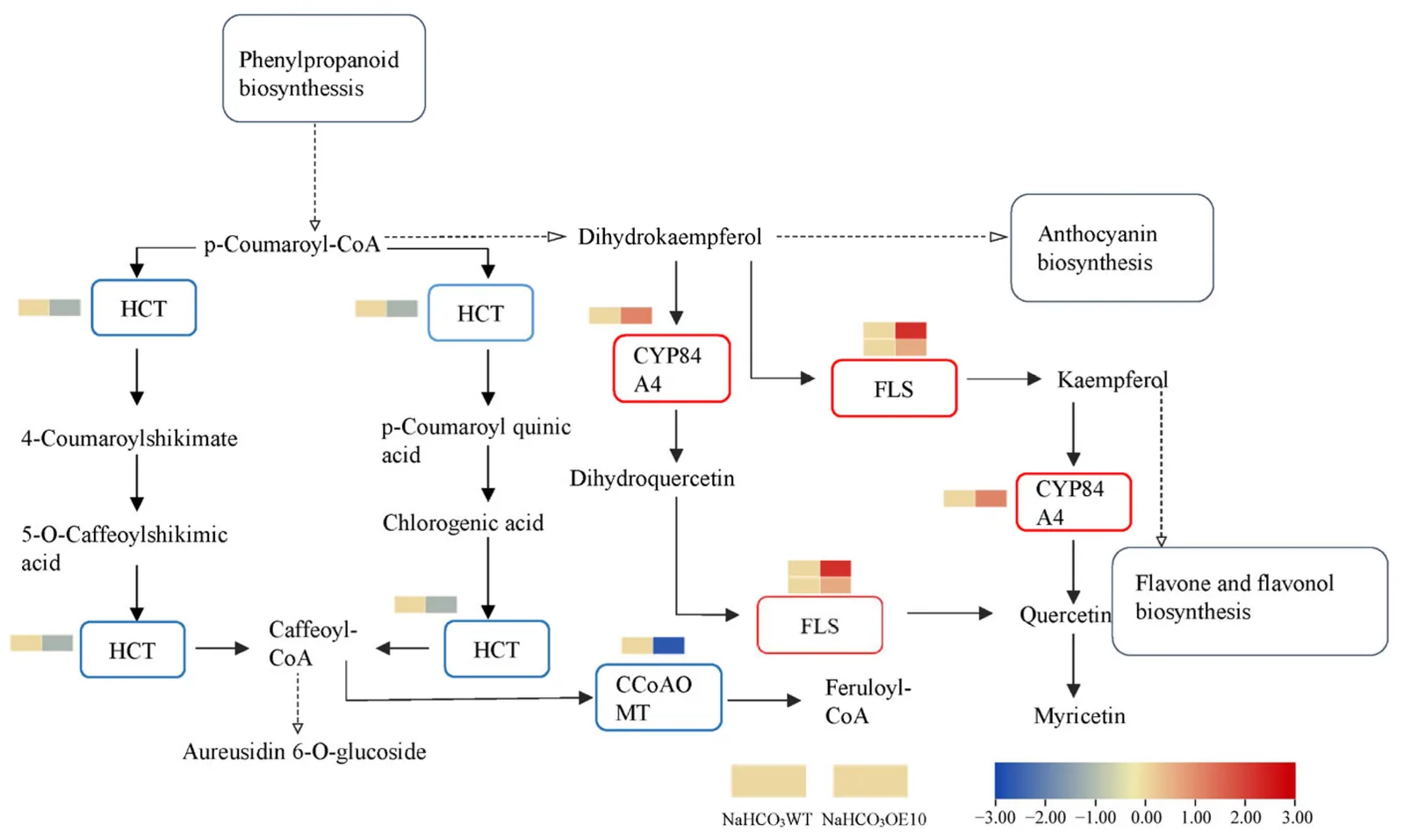
Expression of DEGs related to the flavonoid pathway. HCT, hydroxycinnamoyl-CoA:shikimate hydroxycinnamoyltransferase; CCoAOMT, caffeoyl-CoA O-methyltransferase; CYP75B1, cytochrome P450 superfamily protein; FLS, flavonol synthase.

**Figure 11:**
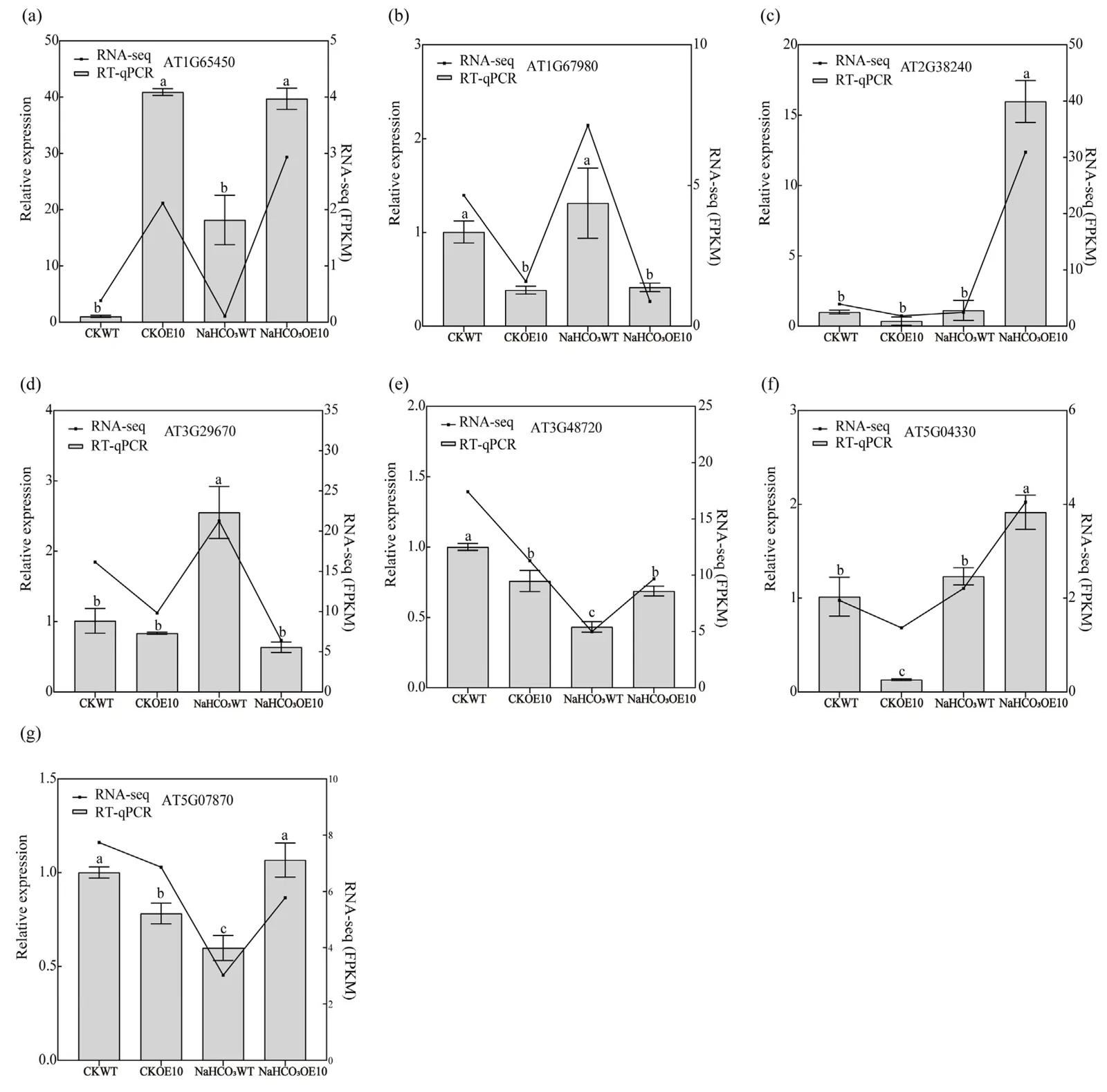
(**a**–**g**) Validation of gene expression from RNA-seq results by qPCR. The left y-axis represents relative expression level, and the right y-axis represents RNA-seq (FPKM) values. The genes corresponding to each panel (**a**–**g**) are as follows: (**a**) At1G65450, *AtGLC*; (**b**) At1g67980, *AtCCoAOMT*; (**c**) At2G38240, *AtJOX4*; (**d**) At3G29670, *AtPMAT2*; (**e**) At3G48720, *AtDCF*; (**f**) At5G04330, At*CYP84A4*; (**g**) AT5G07870 (encoding a putative HXXXD-type acyl-transferase family protein). Data are presented as the mean ± standard deviation (SD) of three biological replicates. Different lowercase letters above the bars indicate significant differences among the groups (*p* < 0.05). This figure was plotted using GraphPad Prism 10; the significance analysis for letter labeling was performed using one-way analysis of variance followed by Duncan’s multiple range test, completed with IBM SPSS Statistics 20.

We performed GO analysis to study the biological functions of the DEGs identified in NaHCO_3_-treated OE10 and NaHCO_3_-treated WT plants. Many of these DEGs were significantly enriched in biological processes such as response to abiotic stimulus, response to oxygen-containing compounds, transmembrane transporter activity, oxygen acid and organic acid metabolism, and response to jasmonic acid (Figure 9c). In the KEGG pathway analysis, the DEGs were mainly enriched in phenylpropanoid biosynthesis, oxidative phosphorylation, photosynthesis, and flavonoid metabolism (Figure 9b). The flavonoid pathway is the most important and major branch of the phenylpropanoid pathway. These two pathways are closely related and together form the core network of plant secondary metabolism. Therefore, we analyzed the expression of genes related to the flavonoid pathway (Figure 10). In the flavonoid biosynthetic pathway, two *FLS* genes and one *CYP75B1* gene were upregulated. In addition, transcriptome data showed that the upstream gene *4CL3*, which can specifically participate in flavonoid biosynthesis, was also upregulated. In contrast, in the lignin biosynthesis branch, which competes with the flavonoid pathway for common precursors, the key genes *HCT*, *CCoAOMT*, *CAD*, and *CCR* were downregulated. To verify the reliability of the RNA-seq data, we randomly selected six differentially expressed genes from the transcriptome data for qRT-PCR analysis. The results showed that the expression trends of qRT-PCR were consistent with those of RNA-seq (Figure 11), confirming the reliability of our data.

## 3. Discussion

Globally, saline–alkali stress not only restricts plant growth and development and crop yield, threatening food security, but also impairs the diversity, stability, and sustainable development of ecosystems [29]. The improvement and utilization of saline–alkali soil currently comprise three categories: physical measures, chemical measures, and biological measures. Among these, physical and chemical measures have the problems of single effects, short duration, and the potential to cause secondary pollution [30]. Phytoremediation is a cost-effective and environmentally friendly technique, widely recommended for reclaiming salinized land into agricultural land by improving soil quality [31,32]. Accordingly, biotechnological approaches and breeding saline–alkali-tolerant varieties are the primary methods and breakthroughs for addressing plant saline–alkali stress and achieving phytoremediation. Elucidating the adaptive mechanisms of saline–alkali-tolerant plants such as sorghum in response to saline–alkali stress and deciphering the regulatory networks and metabolic pathways involving stress-responsive genes are of great practical significance for breeding saline–alkali-tolerant crops and for the development and utilization of saline–alkali lands.

Recent studies have shown that many saline–alkali-tolerant plants have been found to be useful for remediating saline–alkali soil, and the molecular mechanisms and valuable candidate genes have also been uncovered. Studies have demonstrated that the MYB transcription factor family is large and involved in regulating plant responses to biotic and abiotic stresses, as well as in development, differentiation and defense. This family exerts key regulatory roles in saline–alkali stress responses, and an increasing number of members of the MYB gene family have been identified and verified to improve plant tolerance to saline–alkali stress [33,34]. *OsMYB2* encodes a stress-responsive MYB transcription factor and plays a regulatory role in rice tolerance to salt, cold, and dehydration stress [35]. Studies have found that several key MYB family members screened from rice, including *OsMYB91*, *OsMYB103*, and *OsMYB124*, respond to multiple hormones and participate in responses to abiotic stresses such as salt and high temperature [36]. In soybean, *GmMYB68*-overexpressing plants have a higher tolerance to saline–alkali stress than wild-type plants; under saline–alkali stress, they produce significantly more seeds per plant and have a higher 100-seed weight than wild-type plants [37]. In the saline–alkali-tolerant *Sorghum bicolor LTR108*, when under severe saline–alkali stress, MYB transcription factors are preferentially upregulated in *LTR108*, which enhances the tolerance of *LTR108* to saline–alkali stress [38]. Among the MYB gene family, *SbMYB38* encodes an R2R3-type MYB transcription factor. Its N-terminus contains two imperfect repeat sequences (R2 and R3), each approximately 52 amino acids in length, which fold into three α-helices. The second and third α-helices form a helix-turn-helix (HTH) structure. Each repeat contains three regularly spaced, conserved hydrophobic residues that provide a hydrophobic core for HTH folding. This N-terminal region forms a conserved DNA-binding domain. The C-terminal regulatory region affects the direction of transcriptional regulation, target gene specificity, and interactions with other factors. To further clarify the relationship between *SbMYB38* and known MYB family members, a phylogenetic tree was constructed in this study (Figure 1a). The results showed that *SbMYB38* formed an independent clade in the phylogenetic tree, which was parallel and separate from the clade containing maize *ZmMYB42* and *ZmMYB38 (Zea mays)* and sugarcane *ScMYB1* (*Saccharum* spp.). Maize *ZmMYB42* and *ZmMYB38* are R2R3-MYB transcription factors that contain an EAR repression motif and function primarily as transcriptional repressors of phenylpropanoid metabolism [39]. The phylogenetic independence of *SbMYB38* from these known repressor-like members suggests that its sequence has undergone lineage-specific differentiation. Its C-terminal regulatory region may harbor regulatory motifs distinct from those of classical repressors, thereby altering its transcriptional regulatory direction or target gene specificity. Furthermore, the C-terminal regulatory regions of different MYB members vary considerably and may carry activation or repression motifs, which may lead them to exert different or even opposite regulatory roles under the same stress, even though they belong to the R2R3 type [40]. Therefore, *SbMYB38* may possess a regulatory mode distinct from the known *ZmMYB42* and *ZmMYB38*, but its specific function still requires further verification. Currently, there are no reports on the physiological response and molecular mechanism of the *SbMYB38* gene under saline–alkali stress. Previously, we conducted transcriptome studies and found that the *SbMYB38* gene was induced under saline–alkali stress in the Hongyingzi cultivar of sorghum (*Sorghum bicolor*). In this study, we heterologously overexpressed *SbMYB38* in transgenic *Arabidopsis thaliana* to investigate its function in response to saline–alkali stress.

In plants, ROS are normal byproducts of various metabolic processes. They play important roles in biological processes such as plant growth and development, programmed cell death, stress resistance, and hormone signaling [41]. At low concentrations, reactive oxygen species are part of signal transduction processes. When under stress, ROS act as second messengers and regulate the expression of various stress genes by activating different transcription factors [41,42]. However, excessive ROS can cause plant damage, leading to changes in cell structure, degradation of biomolecules, and disruption of various important physiological processes [43]. Therefore, plants must maintain ROS homeostasis in order to grow and develop normally. Plant cells respond to oxidative stress through endogenous defense mechanisms, namely enzymatic and non-enzymatic mechanisms, to remove reactive oxygen species [44]. Malondialdehyde (MDA) is a key product in the process of membrane lipid peroxidation. The content of MDA increases as the degree of oxidation or oxidative damage increases [45]. Proline (Pro) is a substance involved in osmotic regulation and prevents peroxidation [46]. SOD, POD, and CAT are important scavengers and components of the plant antioxidant defense system [47]. In this study, transgenic *Arabidopsis thaliana* plants overexpressing *SbMYB38* exhibit stronger antioxidant capacity and higher antioxidant enzyme activity under sodium bicarbonate stress (Figure 6). The MDA content in the transgenic lines is lower than that in the wild type, indicating that the wild-type lines are more severely affected by peroxides. Additionally, overexpression of *SbMYB38* increases root growth and seed germination rate (Figure 5a,b).

Under saline–alkali stress, high concentrations of sodium ions disrupt the ion balance inside plant cells, leading to damage to the cell membrane, abnormal cell metabolism, and ion toxicity [48]. Plants mainly excrete sodium ions from cells and use ion antiporters such as *SOS1* to alleviate the toxicity of sodium ions [49]. H^+^-ATPase produces a proton driving force. This force drives the transport of sodium ions. More H^+^ are pumped into the cell and the vacuole, which increases the transmembrane electrochemical gradient; as a result, sodium ions flow out of the cell and into the vacuole [50,51]. When a large amount of Na^+^ flows into the cytoplasm, it may cause the membrane potential to drop below the resting potential, activating the K^+^ efflux channels *SKOR*, *NSCC*, and *GORK* [52]. Meanwhile, some K^+^ channels, such as high-affinity K^+^ transporters (HKTs), are involved in plant K^+^ downregulation [53]. Thus, some ion transport genes play important roles in plant responses to saline–alkali stress. To investigate the effect of *SbMYB38* overexpression on ion transport-related gene expression, we examined several genes involved in proton, Na^+^, and K^+^ transport. *SbMYB38* overexpression altered the expression levels of proton pump-, Na^+^-, and K^+^-transport-related genes (Figure 8), resulting in distinct changes: under 150 mM NaHCO_3_ treatment, transcript levels of *AtAHA2* and *AtSOS1* were significantly higher in transgenic lines than in wild-type plants, whereas transcript levels of *AtHKT1*, *AtNHX1*, and *AtSKOR1* were significantly lower. *AtAHA8* was upregulated under saline–alkali stress but showed no significant difference between transgenic and wild-type plants. These expression changes represent the response characteristics of ion transport-related genes in *SbMYB38*-overexpressing plants under saline–alkali stress.

To further investigate the role of *SbMYB38* under saline–alkali stress, we performed GO analysis. The results showed that the DEGs were categorized into biological processes such as response to abiotic stimuli, response to oxygen-containing compounds, transmembrane transporter activity, oxygen-containing acid and organic acid metabolism, and response to jasmonic acid (Figure 9c). KEGG analysis showed that the DEGs were mainly enriched in the biosynthesis of phenylpropanoids, the metabolic pathways of the flavonoid pathway, oxidative phosphorylation, and the photosynthesis pathway (Figure 9b). In addition, the DEGs were also enriched in tryptophan metabolism, linoleic acid metabolism, and the biosynthesis of cutin, suberin, and wax. Flavonoids are a large and diverse group of polyphenolic secondary metabolites in plants and they are synthesized through a specific branch of the phenylpropanoid pathway. Flavonoids can scavenge ROS, protect cells from oxidative damage, and help plants resist saline–alkali stress to some extent [54]. For example, under alkaline stress, flavonoids such as naringin, kaempferol, and quercetin derivatives accumulate in alfalfa, which enhances the antioxidant activity of the plant [55]. Under saline–alkali stress, the flavonoid content in sorghum increases, and the content shows dynamic changes [56]. Flavonoids are also involved in regulating plant hormone signaling pathways, such as those of auxin, jasmonic acid, and abscisic acid, in response to saline–alkali stress [57,58].

The phenylpropanoid pathway is one of the core secondary metabolic regulatory pathways in plant responses to abiotic stress. Its downstream section comprises two major competing branches: flavonoid biosynthesis and lignin biosynthesis. These two branches share precursors such as cinnamoyl-CoA and compete for a common metabolic flux, forming a dynamic equilibrium within the metabolic flux [59,60]. In flavonoid biosynthesis, flavonol synthase (FLS) is a key enzyme in the flavonol biosynthetic pathway in plants. It converts dihydroflavonols including dihydrokaempferol, dihydroquercetin, and dihydromyricetin into kaempferol, quercetin, and myricetin, respectively, and is widely involved in plant abiotic stress responses [61]. Quercetin is a potent antioxidant that improves plant tolerance to multiple abiotic stresses [62]. Intracellular H_2_O_2_ concentration can directly affect the process of stomatal closure, and quercetin can reduce H_2_O_2_ levels, thereby enhancing the ability of plants to cope with various stresses [63,64]. Under drought stress, the levels of flavonoids such as kaempferol and quercetin increase in tomato plants, accompanied by improved drought resistance [65]. Previous studies have shown that 4-coumarate CoA ligase 3 (4CL3), a member of the *Arabidopsis thaliana* 4CL family, specifically participates in flavonoid biosynthesis [66]. Cytochrome P450 super-family protein (CYP75B1) catalyzes flavonoid-related reactions, acting on substrates such as dihydroquercetin, thereby promoting the production of phenolic metabolites and improving abiotic stress tolerance in plants [67]. In plants, hydroxycinnamoyl-CoA shikimate hydroxycinnamoyltransferase (HCT) and caffeoyl-CoA O-methyltransferase (CCoAOMT) are key enzymes in lignin biosynthesis, while cinnamyl alcohol dehydrogenase (CAD) is one of the final key enzymes in lignin monomer synthesis, catalyzing monolignol formation [68,69]. It has been reported that *Arabidopsis thaliana* mutants defective in CAD and cinnamoyl-CoA reductase (CCR) show reduced lignin content in stems but accumulate higher levels of phenolic compounds, including flavonol glycosides, indicating a redirection of phenolic metabolism from lignin biosynthesis in xylem toward flavonoid biosynthesis [70]. Consistent with this metabolic redirection, our transcriptome data revealed that key lignin biosynthetic genes *HCT*, *CCoAOMT*, *CAD* and *CCR* were differentially expressed and all downregulated, whereas key flavonoid biosynthetic genes *FLS*, *4CL3*, and *CYP75B1* were differentially expressed and all upregulated (Figure 10). This expression pattern suggests that *SbMYB38* may be involved in regulating metabolic partitioning between flavonoid and lignin biosynthesis, may contribute to the accumulation of flavonoids such as flavonols, quercetin, and kaempferol, and may thereby enhance the ability of plants to respond to saline–alkali stress. Nevertheless, the current evidence is mainly at the transcriptome level. This hypothesis still requires further experimental validation, and the underlying regulatory mechanism remains to be elucidated.

This study has some limitations. Gene function was characterized via heterologous overexpression in *Arabidopsis thaliana*, and the native function of *SbMYB38* in sorghum under saline–alkali stress still requires further confirmation. Current evidence is limited to transcript-level analysis, lacking Western blot verification of protein accumulation in overexpression lines. Quantitative detection of flavonoid metabolites was not performed; therefore, the regulatory role of *SbMYB38* in the flavonoid pathway at the metabolic level remains speculative. In addition, the differentially expressed genes identified by transcriptome analysis are regarded as transcriptional changes accompanying *SbMYB38* overexpression, and their direct regulatory relationships still require further experimental confirmation. Future work will incorporate protein detection and metabolomic analysis to systematically elucidate the molecular mechanism of *SbMYB38* in saline–alkali tolerance.

## 4. Materials and Methods

### 4.1. Plant Cultivation and Treatment

The materials used in this experiment were *Sorghum bicolor* ‘Hongyingzi’, *Arabidopsis thaliana* (Columbia wild type), and *Nicotiana benthamiana* seeds, all of which were preserved by our research group. The *Sorghum bicolor* ‘Hongyingzi’ seeds were sterilized with 2% sodium hypochlorite for 8 min, rinsed with sterile water five times, and then placed on moist filter paper. They were cultured in a light culture room (28 °C, 16 h light/8 h dark) until they grew into sprouts of 0.3–0.5 cm. The seedlings were then transplanted into half-strength Hoagland solution and grown until they reached the stage of two leaves and one growing point. Twelve seedlings with similar growth were selected and divided into two groups. They were treated for 24 h in half-strength Hoagland solution with or without 150 mM NaHCO_3_, with three biological replicates in each group. The seeds of *Arabidopsis thaliana* were washed with 75% anhydrous ethanol for 2 min, then with 2% sodium hypochlorite for 7 min, and finally rinsed with sterile water three times. They were placed in a refrigerator at 4 °C for vernalization for 3 days and then germinated vertically on Murashige and Skoog (MS) medium for seven days. We transplanted seedlings of wild-type *Arabidopsis* and three transgenic *Arabidopsis* lines into soil pots, with four seedlings of the same line planted in each pot. The plants were grown for 2 weeks in a growth chamber maintained at 20 ± 2 °C, 70% relative humidity, and a 16 h light/8 h dark cycle. Subsequently, plants of the wild type and the three transgenic lines with uniform growth were selected for subsequent stress treatment. From these, one pot of the wild-type line and one pot of each transgenic line were selected, and plant phenotypes were photographed and recorded. The selected plants of each line were subjected to different treatments, with a control group and a treatment group. The control group received 1 L of half-strength Hoagland nutrient solution, and the treatment group received 1 L of half-strength Hoagland nutrient solution containing 150 mM NaHCO3 (PH = 8) For each treatment, one biological replicate contained all different lines, with three pots per line. Three biological replicates were performed. After 7 days of treatment, phenotypes were observed and recorded. Fresh leaves were collected for chlorophyll content measurement, and samples from each line were snap-frozen in liquid nitrogen for storage. The same washing method was used for *Arabidopsis* seeds, which were then placed on half-strength MS medium with or without 6 mM NaHCO_3_ to measure root length and germination rate. Tobacco seeds were directly sown in square boxes filled with moist nutrient soil for germination. When the tobacco seedlings reached the two-leaf stage, they were transplanted individually into square boxes filled with nutrient soil. These plants were grown in a greenhouse, with a temperature maintained at 20 ± 2 °C, 70% humidity, and a 16 h photoperiod.

### 4.2. Phylogenetic Analysis

We translated the CDS sequence of *SbMYB38* into the corresponding amino acid sequence. Using this protein sequence as the query sequence, a BLASTP search was performed in the NCBI protein database. Homologous MYB protein sequences from different species were screened and downloaded, and a total of 11 MYB protein sequences were obtained for phylogenetic analysis. The gene names are *DoMYB* (OEL35709.1), *PvMYB* (XP039815691.1), *SvMYB*(XP034599392.1), *PvMYB4d*(RLN04826.1), *PvMYB4e* (AEM17352.1), *ZmMYB38*(XP008664600.1), *ScMYB*(CCU64153.1), *PaMYB* (XP062196436.1), *OgMYB*(XP052163965.1), *ZmMYB42*(NP001106009.2), and *ObMYB* (XP040382751.1). The content in parentheses is the accession number. The corresponding species in order are *Dichanthelium oligosanthes*, *Panicum virgatum*, *Setaria viridis*, *Panicum miliaceum*, *Panicum virgatum*, *Zea mays*, *Saccharum hybrid* ‘*cultivar Co 86032*’, *Phragmites australis*, *Oryza glaberrima*, *Zea mays*, and *Oryza brachyantha*. The Sequence Identities of *DoMYB*, *PvMYB*, *SvMYB*, *PvMYB4d*, *PvMYB4e*, *ZmMYB38*, *ScMYB*, *PaMYB*, *OgMYB*, *ZmMYB42* and *ObMYB* compared with *SbMYB38* are 79.59%, 77.64%, 75.90%, 78.9%, 76.86%, 81.22%, 89.73%, 75.52%, 73.93%, 77.2%, and 73.31%, respectively. Multiple sequence alignment of the 12 MYB protein sequences was carried out using DNAMAN 5.2.2 software with the default parameters. The alignment results were imported into MEGA 7.0 software, and an unrooted phylogenetic tree was constructed using the Maximum-Likelihood (ML) method (model: Jones–Taylor–Thornton). Bootstrap test was set to 1000 replicates to evaluate the confidence of each branch of the phylogenetic tree, and the remaining parameters were kept as software defaults. The phylogenetic tree was finally exported.

### 4.3. Subcellular Localization

Using the CDS sequence of the *SbMYB38* gene and the pROKII vector containing restriction enzyme sites, primers were designed. The pROKII-SbMYB38-GFP vector was constructed by homologous recombination and then transferred into Agrobacterium GV3101 using the freeze–thaw method. The prepared bacterial solution was introduced into tobacco plants [71]. The tobacco seedlings were placed in an artificial climate chamber for dark treatment for one day, followed by light treatment for two days. The treated leaves were cut with a blade into small pieces of 1 cm^2^, made into temporary slides, and observed and photographed using a laser confocal microscope (Leica; Wetzlar, Hessian, Germany). The pROKII empty vector was used as a control.

### 4.4. RNA Extraction and qRT-PCR

The extracted RNA was reverse-transcribed into cDNA using the All-in-One First-Strand Synthesis MasterMix (with dsDNase) kit (Yugong Biotech, Lianyungang, China), and the cDNA was stored in a −20 °C refrigerator. qRT-PCR was performed using a fluorescence polymerase chain reaction (PCR) detection system (BIOER, Hangzhou, China). Primer sequences were designed at NCBI (https://ncbi.nlm.nih.gov/nucleotide/, accessed on 17 July 2025) and listed in Table S1. The 20 μL reaction system contained 10 μL Taq Visual SYBR qPCR Premix (iScience, Lianyungang, China), 1 μL of forward primer and 1 μL of reverse primer (10 μM for each primer), and 8 μL of cDNA at a concentration of 60 ng/mL. The thermal cycling program consisted of an initial holding stage at 95 °C for 30 s; a cycling stage with 40 cycles of 95 °C for 10 s and 60 °C for 10 s (fluorescence signal acquisition enabled); and a melting-curve stage of 95 °C for 15 s, 60 °C for 1 min, and 95 °C for 15 min (continuous data sampling). For qRT-PCR, three independent biological replicates were performed, with no technical replicates. The relative gene expression levels were calculated as the mean of these three biological replicates, and all statistical analyses were based on these replicates (*n* = 3). Amplification efficiencies ranged from 90% to 110%, and the relative gene expression levels were calculated using the 2^−ΔΔCt^ method [72]. All primers used in this study ae listed in Table S1.

### 4.5. Construction of Arabidopsis thaliana Expression Vectors and Genetic Transformation

Through homologous recombination, the CDS sequence of *SbMYB38* was ligated into pBI121 containing a BamHI restriction site. The recombinant expression vector pBI121-SbMYB38 was then transformed into GV3101. *Agrobacterium* GV3101-mediated genetic transformation was performed using *Arabidopsis* plants grown to the flowering stage [73]. Positive plants were screened on half-strength MS medium containing 50 mg/L kanamycin, and T3 lines were obtained for subsequent studies.

### 4.6. Phenotypic Analysis and Determination of Physiological Indices

Fresh leaves were sampled to measure chlorophyll content. Samples of each line stored in liquid nitrogen were used to determine physiological indices. The kits used for physiological measurements were purchased from Solarbio Science & Technology Co., Ltd. (Beijing, China) and were used to measure superoxide dismutase (SOD; catalog no. BC5160), peroxidase (POD; catalog no. BC0090), catalase (CAT; catalog no. BC0200), malondialdehyde (MDA; catalog no. BC0020), proline (Pro; catalog no. BC0290), and chlorophyll content (catalog no. BC0990). DAB and NBT (RHAWN, Shanghai, China) staining were used to detect the accumulation of H_2_O_2_ and O_2_^−^, respectively. First, staining solutions were prepared: DAB staining solution (1 mg/mL) was prepared by dissolving 0.1 g DAB in 100 mL PB buffer and adjusting the pH to 3.8; NBT staining solution (1 mg/mL) was prepared by dissolving 0.1 g NBT in 100 mL HEPES buffer. Leaves at the same leaf position from NaHCO_3_-treated and control plants (10–12 leaves per treatment) were collected and immersed in the respective staining solutions. DAB staining was performed under light, whereas NBT staining was performed in darkness, with incubation at room temperature for approximately 12 h. After staining, the staining solution was discarded, and 95% ethanol was added for decolorization in a boiling water bath for 5–20 min (with the solution changed during the process) until chlorophyll was completely removed. Finally, the leaves were preserved in 95% ethanol and photographed for documentation.

### 4.7. RNA Sequencing Analysis

Three independent transgenic lines were generated and verified via molecular characterization. All three lines displayed enhanced saline–alkali tolerance relative to the wild type. Among them, the OE10 line exhibited the highest transcript abundance of the target gene and showed the most pronounced improvements in physiological indices under stress. Therefore, transcriptome sequencing was performed on WT and *SbMYB38*-overexpressing *Arabidopsis* OE10 plants treated with 0 mM and 150 mM NaHCO_3_. RNA-seq libraries were sequenced by Majorbio Bio-Pharm Technology Co., Ltd. (Shanghai, China). Three biological replicates were set for each sample. TAIR10 (https://plants.ensembl.org/Arabidopsis_thaliana/Info/Index) (accessed on 17 July 2025) was used as the reference genome. The library was constructed by the company using Illumina^®^ Stranded mRNA Prep (Illumina, San Diego, CA, USA), Ligation. Statistical methods were applied to calculate the base distribution and quality fluctuation of each cycle of all sequencing reads. We performed sequencing quality assessment on the raw sequencing data of each sample, including the statistics of A/T/G/C base content distribution, base quality distribution and base error rate distribution. The raw sequencing data were filtered by the company using fastp (https://github.com/OpenGene/fastp) (accessed on 17 July 2025) to obtain high-quality sequencing data (clean data). The quality-controlled raw data, namely clean data (reads), were aligned to the reference genome using HiSat2 (used by default, http://ccb.jhu.edu/software/hisat2/index.shtml) (accessed on 17 July 2025) to obtain mapped data (reads) for subsequent transcript assembly and expression quantification. Meanwhile, quality evaluation was carried out for the alignment results of this transcriptome sequencing. The expression levels were analyzed using RSEM (used by default, http://deweylab.github.io/RSEM/) (accessed on 17 July 2025), and differentially expressed genes were analyzed by DESeq2 (http://bioconductor.org/packages/stats/bioc/DESeq2/) (accessed on 17 July 2025). The default screening criteria for significantly differentially expressed genes were FDR < 0.05 and |log2FC| ≧ 1. A gene was considered as a differentially expressed gene (DEG) only when it satisfied both of the two conditions at the same time. The sample used for transcriptome sequencing was the *SbMYB38* overexpression line OE10. We made a mistake when submitting sample names to the sequencing company, and the raw sequencing files were incorrectly named NaHCO_3_OE9 and CKOE9. The sequencing reads were actually derived from line OE10, and the correct line designation OE10 is uniformly used for all transcriptome data in this manuscript. The correspondence between raw file names and correct sample names is as follows: NaHCO_3_OE9 corresponds to NaHCO_3_OE10 and CKOE9 corresponds to CKOE10. This correction only concerns sample nomenclature and does not alter the raw sequencing data. The raw sequence data are available under BioProject PRJNA1542195 in the NCBI SRA database.

### 4.8. Statistical Analysis

Data are presented as the mean ± standard deviation (SD) of three biological replicates. Different lowercase letters above the bars indicate significant differences among the groups (*p* < 0.05). Figures were plotted using GraphPad Prism 10; the significance analysis for letter labeling was performed using one-way analysis of variance followed by Duncan’s multiple range test, completed with IBM SPSS Statistics 20.

## 5. Conclusions

In this study, we found that the sorghum gene *SbMYB38* was located in the nucleus. It was highly expressed in roots and stems under saline–alkali stress. To determine the function of *SbMYB38*, we overexpressed this gene in *Arabidopsis thaliana*. The results of antioxidant enzyme activity, NBT staining, and DAB staining showed that, compared with the control, the *SbMYB38* overexpression lines exhibited reduced oxidative damage. This indicates that the *SbMYB38* overexpression lines are tolerant to saline–alkali stress. In addition, overexpression of *SbMYB38* may have regulated the expression of ion transport-related genes such as *AtAHA2*, *AtSOS1*, *AtSKOR1*, and *AtNHX1*, which may have helped reduce ion toxicity under saline–alkali stress. Transcriptome data showed that the key lignin biosynthesis genes *HCT*, *CCoAOMT*, *CAD*, and *CCR* were downregulated, while the key flavonoid biosynthesis genes *FLS*, *4CL3*, and *CYP75B1* were upregulated. This suggests that plants overexpressing *SbMYB38* may display tolerance to saline–alkali stress at both physiological and biochemical levels, possibly by increasing antioxidant enzyme activity and regulating the expression of genes involved in the flavonoid biosynthesis pathway. These findings provide insights into the potential molecular mechanism underlying plant tolerance to saline–alkali stress and may be valuable for the phytoremediation of saline–alkali land. At present, the area of salinized land continues to expand, which severely restricts agricultural development. Future studies may identify saline–alkali-tolerant genes in crops and apply transgenic technology to improve these crops, thereby breeding varieties with enhanced saline–alkali tolerance.

## Data Availability

The datasets utilized in this work are available upon reasonable request.

## Supplementary Materials

The following supporting information can be downloaded at https://www.mdpi.com/article/10.3390/plants15213283/s1: Table S1. Primers for qRT-PCR analysis in this study. *SbActin*, the reference gene for *Sorghum bicolor*; *AtActin*, the reference gene for *Arabidopsis thaliana*.
