# Supplementary material for: Overexpression of SbMYB38 from Sorghum bicolor Enhances Saline–Alkali Stress Tolerance in Arabidopsis thaliana
Source: Plants (Basel). 2026 Oct 24;15(21):3283. doi: 10.3390/plants15213283 (PMC13765874; doi:10.3390/plants15213283)
Supplement: Supplementary file 1 [file plants-15-03283-s001.zip › plants-4577529-supplementary.pdf]

Table S1. Primers for qRT-PCR analysis in this study. *SbActin*, the reference gene for *Sorghum bicolor*; *AtActin*, the reference gene for *Arabidopsis thaliana*

Gene Name	Primer Name	Primer Sequence (5′-3′)
AtAHA2	F	GCCATAACTCTTCCCAAACCTTA
	R	AGAAGCCGGAAGAAAAGTTGG
AtAHA8	F	CCCTATCCAACACCGCACAT
	R	CCTCTTGGTGATGGCACCTT
AtHKT1	F	TCTTCTTGGAGTGACGGTGC
	R	TAGTTTCTCCGGTGTGTCGC
AtNHX1	F	TACGTGGCTTCTTGACACGG
	R	TTCCACGTTACCCTCAAGCC
AtSKOR1	F	CGTTTCCGAGTGGCTCCAAA
	R	TACCCACAGTACAATCCCGC
AtSOS1	F	ACACCACCACGTAGCTCAAG
	R	TGCCGAAAATGCTGAGTTGC
At1G65450	F	AACAAGGAGTTTCCACCGGA
	R	TCCAAGTTCCTCCAACGTGT
At1G67980	F	TTTGCACACAACCACGAGAC
	R	TGACCCTCATCAACCGGAAC
At2G38240	F	CAAACCGCTATGTGAAGCCG
	R	CCATCAACGAGTGGGTCACA
At3G29670	F	CGGTTAGATCCGAAGCTCCC
	R	TCCTTCCACGAAAGTGTCCG
At3G48720	F	GGATATTGCGGGAACGGGAT
	R	AGTGTCGCGTTCATACTCGG
At5G04330	F	TTCTCAAACCGTCCCACCAC
	R	TGGAAGCCACTAAACGGACC
At5G07870	F	TGAAGTCTCCTTTACCGTTGC
	R	CCCGTCATACTTACCGCCAT
SbActin	F	TCTTCCAACCCTACCGCACCA
	R	CGTCATCTCCAGCGAACCCAGCCTT
AtActin	F	CTTAACCCAAAGGCCAACAGA
	R	GCAAGGTCAAGACGGAGGAT
